# Neonatal Seizures—Perspective in Low-and Middle-Income Countries

**DOI:** 10.1007/s12098-021-04039-2

**Published:** 2022-01-20

**Authors:** Hemadri Vegda, Vaisakh Krishnan, Gabriel Variane, Vaishnavi Bagayi, Phoebe Ivain, Ronit M. Pressler

**Affiliations:** 1 Center of Perinatal Neuroscience, Department of Brain Sciences, Imperial College, London, UK; 2 Neonatal Intensive Care Unit, Bangalore Medical College and Research Institute, Bengaluru, Karnataka India; 3 Institute of Maternal and Child Health, Calicut Medical College, Kozhikode, Kerala India; 4 Protecting Brains & Saving Futures, McGill University Health Center/Research Institute of the McGill University Health Center, São Paulo - SP, Brazil; 5 Neonatal Intensive Care Unit, Karnataka Institute of Medical Sciences, Hubbali, Karnataka India; 6 Center for Perinatal Neuroscience, Brain Sciences Department, Imperial College of Science Technology and Medicine, London, UK; 7 Department of Clinical Neurophysiology, Great Ormond Street Hospital for Children NHS Trust, London, UK; 8 Department of Clinical Neuroscience, UCL- Great Ormond Street Institute of Child Health, London, WCIN IEH UK

**Keywords:** Neonatal seizure, Low- and middle-income countries, EEG, Treatment

## Abstract

Neonatal seizures are the commonest neurological emergency and are associated with poor neurodevelopmental outcome. While they are generally difficult to diagnose and treat, they pose a significant clinical challenge for physicians in low- and middle-income countries (LMIC). They are mostly provoked seizures caused by an acute brain insult such as hypoxic–ischemic encephalopathy (HIE), ischemic stroke, intracranial hemorrhage, infections of the central nervous system, or acute metabolic disturbances. Early onset epilepsy syndromes are less common. Clinical diagnosis of seizures in the neonatal period are frequently inaccurate, as clinical manifestations are difficult to distinguish from nonseizure behavior. Additionally, a high proportion of seizures are electrographic-only without any clinical manifestations, making diagnosis with EEG or aEEG a necessity. Only focal clonic and focal tonic seizures can be diagnosed clinically with adequate diagnostic certainty. Prompt diagnosis and timely treatment are important, with evidence suggesting that early treatment improves the response to antiseizure medication. The vast majority of published studies are from high-income countries, making extrapolation to LMIC impossible, thus highlighting the urgent need for a better understanding of the etiologies, comorbidities, and drug trials evaluating safety and efficacy in LMIC. In this review paper, the authors present the latest data on etiology, diagnosis, classification, and guidelines for the management of neonates with the emphasis on low-resource settings.

**Supplementary Information:**

The online version contains supplementary material available at 10.1007/s12098-021-04039-2.

## Introduction

Neonatal seizures are the most common neurological emergency in the neonatal period and often pose diagnostic and management challenges for clinicians across the world [1]. Recently, a number of published studies have been aimed at improving the diagnosis, treatment, and outcome of neonatal seizures. However, the majority of these studies originate from high-income countries (HIC). There is evidence that the incidence and etiologies of neonatal seizures differ in HIC and low- and middle-income countries (LMIC),which may have far-reaching implications for their management [2]. Furthermore, most studies from LMIC solely rely on clinical diagnosis for seizure identification due to the limited availability of electroencephalography (EEG) and amplitude-integrated EEG (aEEG) [2–5]. In this review, we address the diverse manifestations of neonatal seizures, their incidence, etiology, the diagnostic role of aEEG/EEG, evaluation, and management within low resource settings. As high-quality evidence from LMIC is lacking, much of the authors' recommendations are based on the evidence from HIC.

## Definition of Neonatal Seizures

The International League Against Epilepsy (ILAE) defines seizures as a transient occurrence of signs and/or symptoms due to abnormally excessive or synchronous neuronal activity in the brain. This definition excludes the electrographic-only seizures, which constitute a 40%–60% of all seizures occurring in critically ill neonates [6–8]. The American Clinical Neurophysiology Society classifies seizures into clinical-only, electroclinical, or electrographic-only seizures. Electrographic seizures are defined as a paroxysmal abnormal, sustained change in the EEG characterized by a repetitive and an evolving pattern with a minimum 2 µV voltage (peak to peak) and a duration of at least 10 s [9]. "Evolving" means here an unequivocal, gradual change in frequency, amplitude, morphology, and location. This definition is now generally accepted by the experts and has major implications for the diagnosis of seizures, as it requires the availability of aEEG or EEG [2, 10].

## Epidemiology of Neonatal Seizures

The incidence of neonatal seizures is estimated to be 1–3 per 1000 live births in HIC and widely varies from 36–90 per 1000 live births in LMIC (Supplementary Table S1). Reports from LMIC are not only scarce but methodology is variable and seizure detection are mostly reliant on clinical diagnosis [2]. In an extensive literature review for the case definite of neonatal seizures by the Brighton Collaboration [2], only four studies on incidence of neonatal seizures from LMIC were described [3, 4, 11, 12], all of which relied solely on clinical diagnosis (Supplementary Table S1).

## Etiology of Neonatal Seizures

Most of the neonatal seizures are acute provoked seizures indicating that they are secondary to an acute brain injury or systemic insult. Only 10%–15% of seizures in the neonatal period are the first manifestation of an epilepsy syndrome (unprovoked seizures), typically due to an underlying structural or genetic etiology [13]. Hypoxic–ischemic encephalopathy (HIE) remains the most common cause of neonatal seizures. Other causes include perinatal stroke, intracranial hemorrhage, metabolic, and electrolyte disturbances, systemic and central nervous system infections, and inborn errors of metabolism or genetic epilepsy syndromes [14]. A history and physical examination complemented with video EEG/aEEG can aid early diagnosis of the underlying etiology, which is important for management and prognostication.

The etiological spectrum of neonatal seizures is different in HIC and LMIC (Supplementary Table S2). While HIE is the major cause in both HIC and LMIC (although more frequent in LMIC), infections have been reported as the second most common cause of seizures in LMIC [5, 15]. Although intracranial hemorrhage and perinatal stroke have been reported to be less common than infections in the LMIC, it is likely that there is under-diagnosis due to a lack of neuroimaging. Overall, the infection rate is similar in babies with encephalopathy across HIC and LMIC (Supplementary Table S2). Seizures resulting from metabolic abnormalities such as hypoglycemia and kernicterus used to be more common in HIC but are now more common in the LMIC compared to HIC, reflecting the better overall supportive care in the HICs [2]. It is possible that there is relevant comorbidity in LMIC, for example, a neonate with HIE may also have an infection or hypoglycemia, which may impact outcome [16]. However, no study has specifically evaluated the effect of comorbidity on outcome.

## Clinical Presentation

Neonatal seizures are different to seizures in older children in their presentation, with a substantial proportion being electrographic-only, lacking a clinical correlate. Mizrahi and Kellaway studied seizure patterns in 71 neonates using video EEG, 11 of which never showed a clinical correlate [6]. Focal clonic seizures, certain myoclonic seizures, and focal tonic seizures were most reliably associated with an electrographic correlate. Most so-called “subtle seizures”[1] were poorly correlated with EEG seizure activity [6] and are now considered not to be seizures [6, 17]. In another study evaluating neonates with encephalopathy undergoing therapeutic hypothermia with video EEG monitoring, 43% failed to show any clinical event among the 14 infants who developed electrographic seizures [18]. A recent prospective study involving 426 neonates with suspected clinical seizures found that 62% of the subjects had at least 1 electrographic-only seizure and 16% had electrographic seizures without any clinical manifestations [8]. Types of clinical seizures are summarized in Supplementary Table S3 [17].

## Classification

**Fig. 1:**
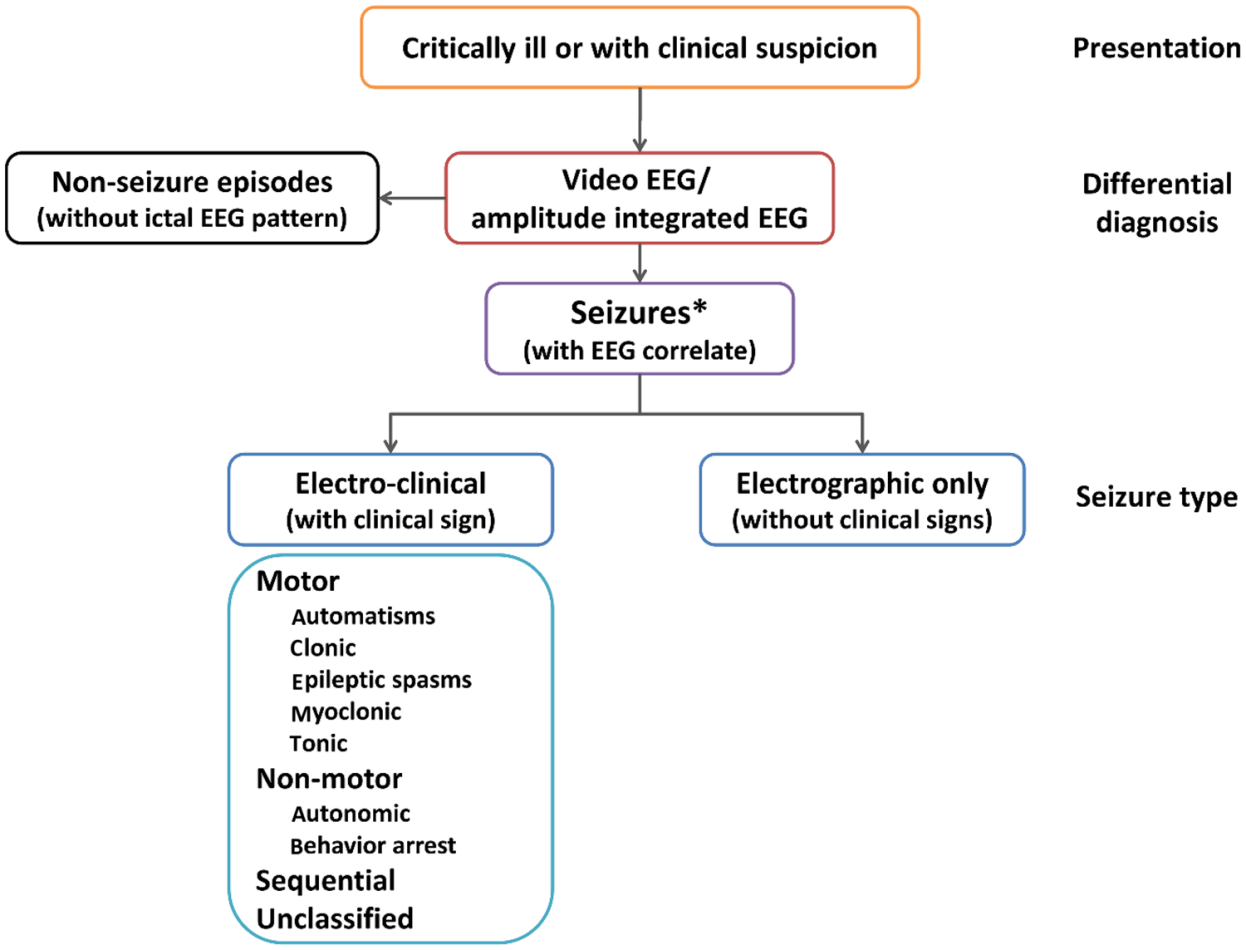
ILAE neonatal seizure classification: diagnostic framework of seizures in the neonatal period including a classification of seizures. *If no EEG available, refer to global alignment of immunization safety assessment in pregnancy levels of diagnostic certainty. Reprinted with permission from [17]

Recently, the ILAE published a new classification of neonatal seizures (Fig. 1) which was adapted from the new classification of seizures for adults and older children [19]. It emphasizes the role of EEG or aEEG for the confirmation of suspected clinical events and is based on an electroclinical relationship of seizures (electroclinical, electrical-only). It then classifies the seizure according to semiology (*motor:* automatisms, focal clonic, focal tonic, myoclonic, epileptic spasms, and *nonmotor:* autonomic and behavioral arrest) each with additional modifiers [17]. Those events which fail to demonstrate a seizure activity in EEG are not considered to be seizures [17, 20, 21].

## Diagnosis of Neonatal Seizures and the Role of EEG/aEEG

EEG is considered the gold standard for the diagnosis of seizures in newborns. However, even in HIC, EEG is not available to many neonatal units and rarely 24/7 due to its time-intensity, expensive equipment and the requirement for expert interpretation [22].

The availability of EEG or even aEEG is limited in most LMIC and consequently there is also little expertise in the interpretation of neonatal recordings. Clinical diagnosis is unreliable due to the risk of both under and overdiagnosis of seizures. Underdiagnosis is due missing discreet seizure manifestations and because over 50% of seizures are electrographic-only while overdiagnosis is due to misdiagnosing abnormal nonepileptic movements as seizures [20]. In another study where 20 video clippings of suspected clinical seizures were evaluated by 91 doctors and 46 other professionals, the average number of events correctly identified was found to be just 10 out 20. The study also found that the evaluators were more likely to identify a clonic seizure correctly as opposed to a subtle seizure [21], thus illustrating how EEG is a necessary tool for seizure detection and clinical diagnosis alone cannot be relied on.

When and if EEG is not available, one- or two-channel amplitude integrated EEG (aEEG) can be helpful for long term monitoring in NICU. A survey from Brazil found half of all neonatal units providing performing therapeutic hypothermia for HIE use to some kind of neuromonitoring, but very few (< 5%) have access to continuous video EEG suggesting that aEEG is the most common form of neuromonitoring [23]. The same group presented evidence how useful aEEG can be in a LMIC setting [24]. aEEG provides an assessment of background activity (which provides information on the degree of brain injury for example in HIE) and can identify seizures. However, the sensitivity of aEEG is lower than full EEG as short seizures (< 30 s) or low amplitude seizures are often missed. There is also a risk of erroneous interpretation of artefacts as seizures and hence the recording must be appropriately annotated. Many new aEEG machines have the option of adding 1–2 raw channels for interpretation which increase the seizure detection rate and identification of artefact. A recent study reported a median sensitivity for aEEG with raw channels of 78% and a median specificity of 78% to detect individual seizures, but without raw traces, the median sensitivity dropped to 54% [25]. Hence, it is recommended that a full-montage video EEG be done whenever possible, or if EEG is not available to use aEEG with raw channels. The American Clinical Neurophysiology Society recommends EEG monitoring for 24 h in all neonates who are at high risk for seizures, such as neonates with acute brain injury, clinical encephalopathy, or abnormal paroxysmal events [26].

In cases where EEG is not available, it is recommended to use the levels of diagnostic certainties as given by the GAIA [2] and subsequently adapted by the ILAE [17]. The five levels of diagnostic certainties are (Supplementary Fig. S1):

1. **Level 1: Gold standard, definite seizure** (Seizures confirmed on EEG with or without clinical manifestations)
2. **Level 2: Probable seizure** (Clinically assessed focal clonic or focal tonic seizures or seizures confirmed on aEEG)
3. **Level 3: Possible seizure** (Clinical events suggestive of epileptic seizures other than focal clonic or focal tonic seizures)
4. **Level 4: Not seizure** (Reported clinical events that do not meet case definition)
5. **Level 5: Not seizure** (Clinical events evaluated by EEG and diagnosed as not a seizure)

## Management of Neonatal Seizure

As with any other neurologic emergency, management of airway, breathing and circulation is of utmost importance. Diagnosis and treatment of underlying etiology is crucial for effective control of neonatal seizures, especially secondary to metabolic derangements. In all suspected seizures, a bedside blood glucose and electrolyte measurement should be done first. Any hypoglycemia must be promptly corrected. Brief seizures secondary to transient metabolic derangements (hypocalcemia, hypoglycemia, hypomagnesemia, or hyponatremia) may not warrant anticonvulsant medication, if seizures cease upon correction. Blood, urine and cerebrospinal fluid analysis with respective cultures should be sent followed by empiric antibiotic therapy, or antiviral therapy, if sepsis is suspected. Any suspicion of a CNS infection must prompt institution of empiric antibiotics or antivirals after taking blood cultures. Supplementary Fig. S2 in the online supporting information illustrates an approach to the evaluation of neonatal seizures.

### Antiseizure Therapy

Experts agree that seizures should be treated as soon as possible while at the same time avoiding any unnecessary use of antiseizure medication [10, 27]. In the absence of evidence, it is recommended that one should commence antiseizure medications when the overall seizure burden is more than 1–2 min on EEG or aEEG [10]. In case EEG is not available, the level of diagnostic certainty must be considered. Focal tonic and focal clonic seizures are most reliably diagnosed, and treatment must be commenced without delay. Timely intervention is crucial as 43% of the neonatal seizures may progress to status epilepticus, if untreated [28, 29].

Since it is assumed that clinical and subclinical seizures differ primarily in anatomical origin, it is important to treat subclinical seizures as well [30]. Clinical manifestations are more likely when the motor cortex is involved. Furthermore, with antiseizure treatment, seizures are more likely to be electrographic-only due to uncoupling. Uncoupling is a phenomenon when the clinical manifestation of seizures subsides, but electrographic seizures persist [30, 31]. GABAergic antiseizure drugs such as phenobarbital induce uncoupling. EEG monitoring helps detect continued electrographic seizures during treatment. Neonates in a deep coma, on heavy sedation or muscle relaxation may also not exhibit clinical manifestations.

### Choice of First- and Second-Line Drugs

Table 1 summarizes the most commonly used antiseizure drugs for the treatment of seizures in the neonatal period. Despite the lack of robust evidence, phenobarbital remains the preferred first-line agent for managing neonatal seizures world-wide [32, 33]. Until recently there was limited evidence for its efficacy [34]. In a randomized controlled trial of first-line therapy phenobarbital vs. phenytoin for EEG-confirmed seizures, phenobarbital was effective in 43% and phenytoin in 45% [35]. The recent NEOLEV2 trial compared the efficacy and safety of phenobarbital and levetiracetam as first-line antiseizure drugs. In this randomized controlled trial, 83 neonates with EEG-confirmed seizures were allocated phenobarbital (*n* = 30) or levetiracetam (*n* = 53); phenobarbital resulted in complete seizure freedom at 24 h in 80% compared to 28% for levetiracetam. Based on the NEOLEV2 study, phenobarbital and not levetiracetam, should be used as the first treatment option for neonatal seizures [33].

**Table 1:** Antiepileptic drugs used in the neonatal period

Medication	Dosage	Common side effects	Remarks
Phenobarbitone	Loading dose: 20 mg/kg intravenously, repeated once as needed (consider 10 mg/kg, if notventilated) Maintenance dose: 3–6 mg/kg/d Target level: 40 mcg/mL	Respiratory depression Depressed consciousness Hypotension Hepatotoxic Blood dyscrasia	Prolonged half-life first week of life and preterm (43–217 h) may lead to increased duration of NICU stay Risk of dose error because of available strength [200 mg/mL] Renal and hepatic excretion can be affected in HIE
Phenytoin/Fosphenytoin	Loading dose: 20 mg/kg PE intravenous, over 20 min or at rate of 3 mg/kg/min PE Maintenance dose: 2.5–5 mg/kg/d in 2 divided doses Target level: 10–20 mcg/mL Administer over 10 min	Infusion site irritation Arrhythmia Rash Hepatotoxic Blood dyscrasia	Cardiac monitoring required Phenytoin poor oral bioavailability Fosphenytoin preferred over phenytoin Levels likely higher in therapeutic cooled infant, and hence, maintenance dose needs to be titrated to drug levels
Levetiracetam	Loading dose: 40–60 mg/kg/d intravenously Maintenance dose: 30–60 mg/kg/d in 3 divided doses Optimal dosing & target level not known	Mild sedation Irritability	Limited information regarding dosing side effect for the neonatal population Adjust dose in renal impairment
Midazolam	Loading dose: 0.15 mg/kg as bolus intravenously over 10 min Maintenance dose: Infusion started at 0.06 mg/kg/h and titrated upwards to effect up to maximal 0.3 mg/kg/h	Respiratory depression Depressed consciousness Hypotension	Developing brain may have an excitatory response to benzodiazepines rather than inhibition, hence, can potentiallyworsen seizures. Wean gradually

*h* Hour; *HIE* Hypoxic–ischemic encephalopathy; *kg* Kilogram; *mcg* Microgram; *mg* Milligram; *min* Minute; *PE* Phenytoin equivalent

When a reversible, acute metabolic etiology for seizures is suspected and investigation results are pending, acute treatment with benzodiazepines with a short half-life (lorazepam, midazolam) may be considered, as the use of phenobarbital may increase the duration of the NICU admission.

**Fig. 2:**
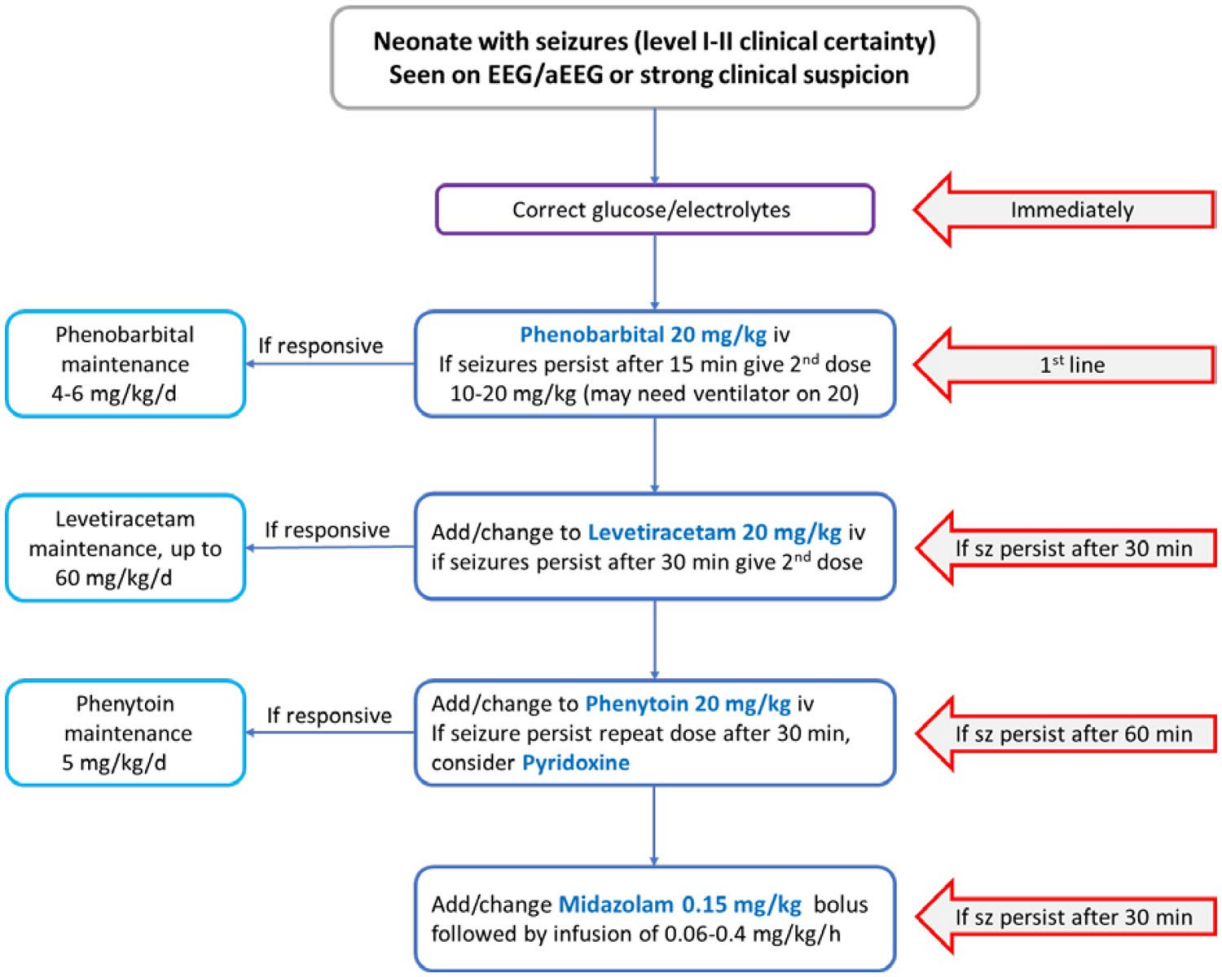
Neonatal seizures treatment algorithm. If no reduction in seizure burden, change to next-line antiseizure drug if clear effect but seizures still ongoing add on next antiseizure drug. *iv* Intravenous; *sz* Seizures

Neonatal seizures which are refractory to phenobarbital typically respond poorly to any second-line antiseizure medication. Evidence for the second-line drug is mainly derived from case series. Phenytoin/Fosphenytoin, levetiracetam, and midazolam may be selected as a second-line antiseizure drug. Phenytoin/Fosphenytoin needs to be given under cardiac monitoring, which may be difficult in some low-resource settings. Levetiracetam is, therefore, considered a better option, but data for its efficacy as a second-line choice are limited. Figure 2 illustrates an example of an algorithm for the treatment of neonatal seizures.

### Treatment Considerations in Inborn Errors of Metabolism

Inborn errors of metabolism represent a particular challenge in LMIC, as early diagnosis is important for timely treatment, assessment of prognosis and genetic counseling [36]. The most important metabolic conditions to consider are pyridoxine/ pyridoxal-p-phosphate–dependent epilepsy, nonketotic hyperglycinemia, molybdenum cofactor deficiency and sulfite oxidase deficiency, mitochondrial disorders, and organic acidurias—urea cycle disorders [36–38].

Pyridoxine dependent seizures manifest early and a therapeutic trial of pyridoxine, pyridoxal 5 phosphate or folinic acid should be considered when seizures are refractory to conventional antiseizure drugs. Availability of intravenous pyridoxine is limited in many LMIC. Often an intravenous multivitamin injection is used instead, but their efficacy and safety has not been evaluated and thus, it is not recommended. Instead, the authors recommend to give 100 mg pyridoxine orally for 3 d. It is important to remember that in rare severe cases, apnea or respiratory arrest may occur with the first 1–2 doses and thus continuous monitoring of vital signs for 2–3 d of treatment initiation is required. A diagnostic algorithm for refractory seizures due to suspected inborn errors of metabolism is given in Supplementary Fig. S3 of the online supporting material.

### Stopping Antiseizure Drugs

**Fig. 3:**
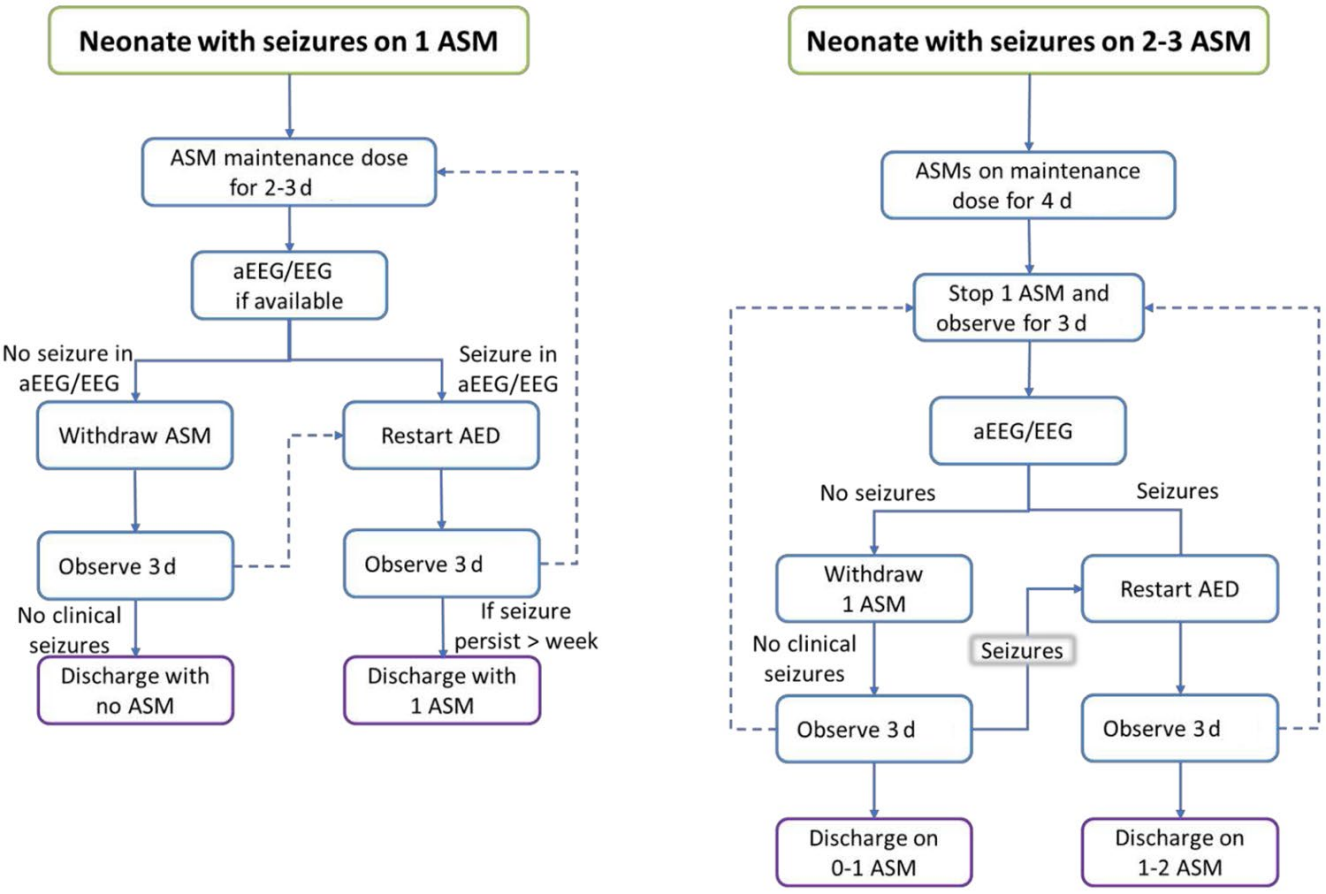
Algorithm for discontinuation antiseizure medication in the neonatal period. If seizures persist for > 7 d, consider discharging on 1–2 AMS with optimized efficacy. ASM antiseizure medication; dashed line: consider action within clinical context

The decision to stop antiseizure medication should be governed by the risk of seizure recurrence. In the case of acute symptomatic seizures, early discontinuation of antiseizure drugs before or shortly after discharge is now generally recommended as this seizure usually resolves within two to three days and the risk of recurrence is low [39]. If seizures were difficult to control, then reducing the number of antiseizure medications to one or two in the neonatal period is preferable and phenobarbital should be the last drug to be discontinued. In a newborn where seizures could not be controlled or newborns with early onset epilepsy, antiseizure medications should be maintained and the newborn should be referred to a pediatrician or child neurologist for the decision to, if, and when to wean medication. A suggested algorithm for stopping antiseizure medication in the neonatal period is shown in Fig. 3.

## The Outcome of Neonatal Seizures

Neonatal mortality associated with seizures has improved drastically over the last three decades, with a reduction in mortality to approximately 7% [40], which can be explained by the improvement of neonatal outcomes in general. In a recent study from a rural district in Kenya involving 142 children with neonatal seizures, 32 died of various causes which amounts to a mortality of 22.5%, indicating that mortality is much higher in LMIC. Regardless of the cause of neonatal seizures, the long-term outcome remains poor. Seizures on their own increase brain injury [41] and a seizure burden of more than 13 min per hour increases the odds of an abnormal neurological outcome by eightfold (OR: 8.00; 95% confidence Interval: 2.06–31.07) [42, 43]. Mortality and morbidity remain high among the preterm neonates with seizure. In a follow-up study from Istanbul, Turkey, of 112 infants with neonatal seizures, 28% of infants later developed cerebral palsy, 36% epilepsy, and almost 50% developmental delay [44]. There is evidence that in neonates with HIE a reduction of electrographic seizure burden guided by aEEG improves cognitive outcome [45, 46]. Early treatment of electrographic seizure burden has been shown to be associated with fewer cases progressing to status epilepticus and a shorter hospital stay [28, 47]. These data indicate that there is a need for improved diagnosis of both electroclinical and electrographic seizure burden to enable earlier treatment.

## EEG Monitoring Systems and the Future in LMICs

In a low-resource setting, telemedicine may play an interesting role in providing remote specialized assistance. Centralized systems can reach a large number of centers in real-time, with educational activities, consultation, and monitoring to leverage the quality of care. After proving the value of aEEG monitoring in a LMIC [24], such a system has successfully been implemented by Protecting Brains and Saving Futures (PBSF) in over 30 hospitals across all regions of Brazil [48]. Babies are monitored with EEG or aEEG and assisted by a team in a remote monitoring center, with encrypted data of EEG transmitted to a secure cloud-based server. This approach is suitable for clinical management or research. However, cost-effectiveness as well as legal and regulatory issues remain important challenges to this approach in LMICs.

## Conclusion

In summary, neonatal seizures pose a number of diagnostic and therapeutic challenge in LMIC:

- Etiology and comorbidity of neonatal seizures specific to the social, economic, and environmental situation in LMIC.
- Availability of EEG and/or aEEG.
- Availability of monitors and ventilators in limited-resource settings (may influence use of maximal therapeutic doses of some antiseizure drugs, such as phenobarbital, phenytoin, or midazolam).
- Availability of infusion pumps and associated risk of drug errors.
- Availability of pyridoxine (IV and oral perpetrations), pyridoxal-5 phosphate, and folinic acid.
- Availability and costs of metabolic and genetic testing.

It is well recognized that some of these concerns are also applicable to neonatal units in high-resourse settings. All of these need to be addressed urgently to improve diagnosis, treatment, and consequently, the outcome of seizures during the neonatal period.

## Supplementary Information

Below is the link to the electronic supplementary material.

Supplementary file1 (DOCX 474 KB)
