# Supplementary material for: Neonatal Seizures—Perspective in Low-and Middle-Income Countries
Source: Indian J Pediatr. 2022 Jan 20;89(3):245–53. doi: 10.1007/s12098-021-04039-2 (PMC8857130; doi:10.1007/s12098-021-04039-2)
Supplement: Supplementary file 1 — Supplementary file1 (DOCX 474 KB) [file 12098_2021_4039_MOESM1_ESM.docx]

**Supplementary Fig. S1** Algorithm to determine degrees of diagnostic certainties for neonatal seizures


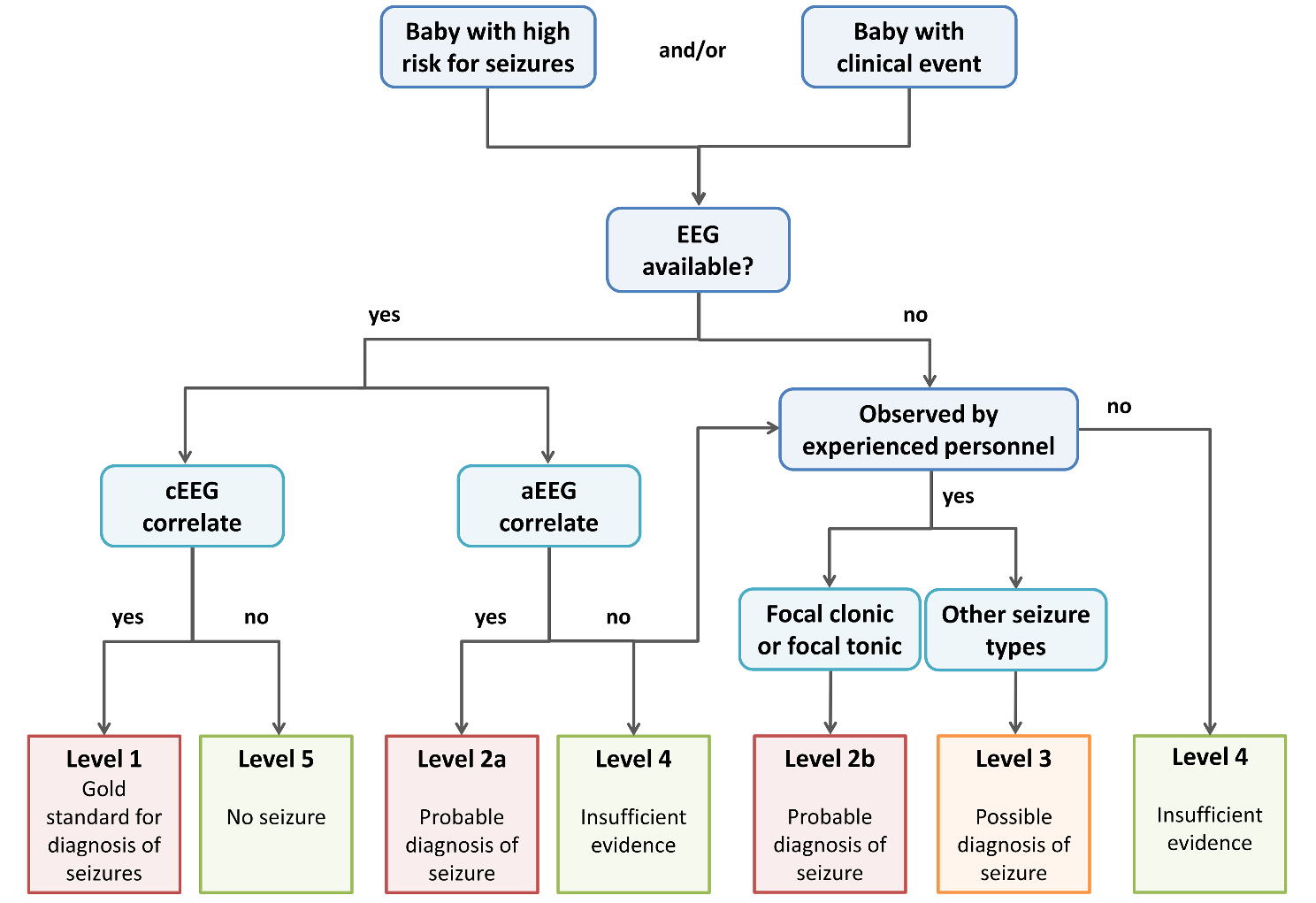


This flowchart will help to determine the diagnostic certainty of neonatal seizures depending on the available diagnostic method (EEG, aEEG or observation by experienced personnel) and seizure type. Developed by the Brighton collaboration^1^ and adapted by the ILAE Neonatal task force. cEEG conventional EEG; aEEG; amplitude-integrated EEG (reprinted with permission from^2^)

**Supplementary Fig. S2** Evaluation of seizures

*ABG* Arterial blood gas; *CSF* Cerebrospinal fluid; HIE Hypoxic ischemic encephalopathy; *MRI/MR* Magnetic resonance imaging/Magnetic resonance; *TMS* Tandem mass spectrometry; *USG* Ultrasound scan

^Next generation sequencing - clinical exome or whole exome sequencing

**Supplementary** **Fig. S3** Algorithm for refractory seizures due to suspected inborn error of metabolism [3, 4].


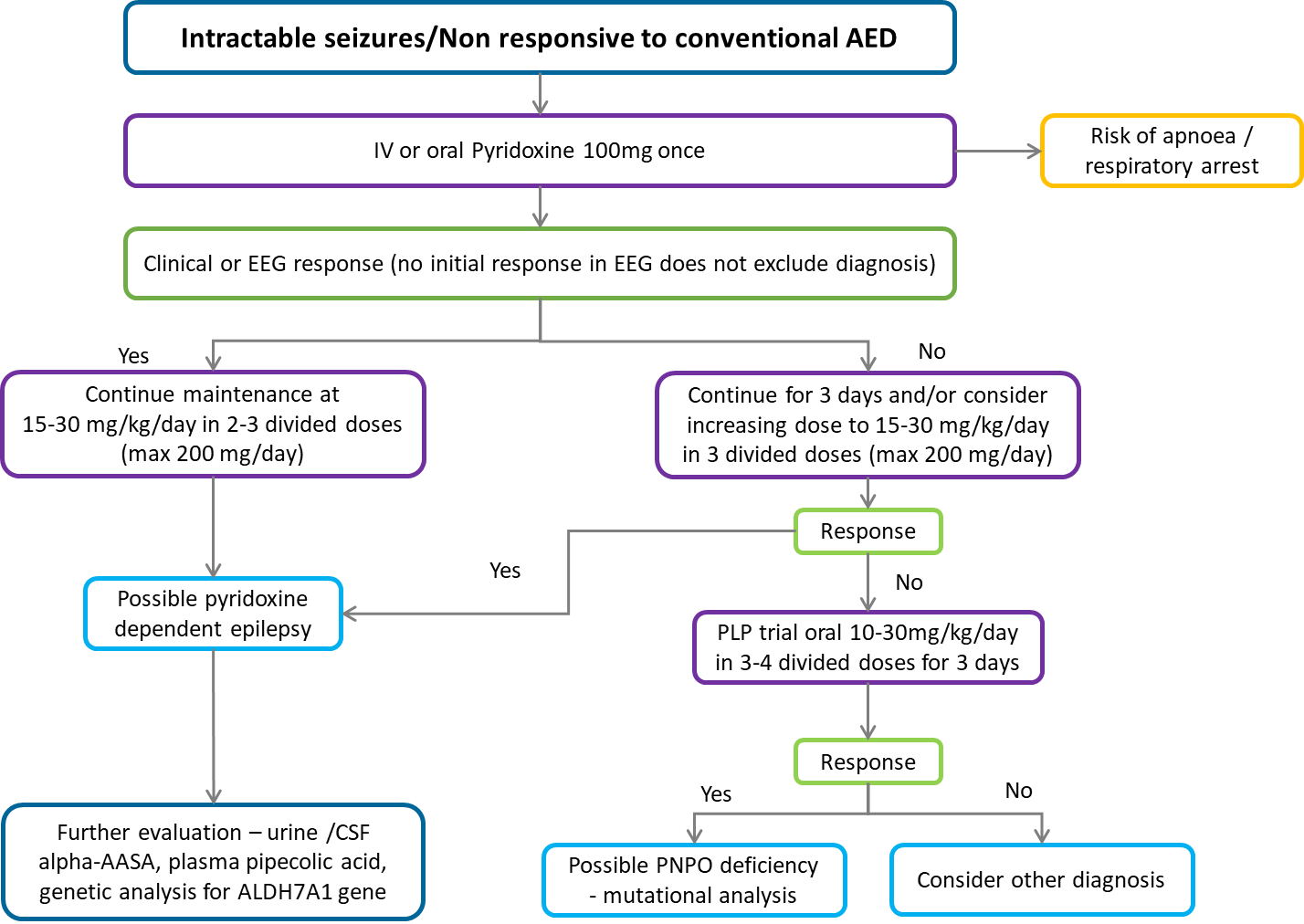


*AASA* Alpha-aminoadipic semialdehyde; *ALDH7A1 or Antiquitin* Aldehyde dehydrogenase 7 family member A1; *PLP* Pyridoxal-5-phosphate; *PNPO* Pyridoxal-5-phosphate oxidase deficiency

**Supplementary Table S1** Incidence of neonatal seizures (A) Incidence per 1000 live births across the HIC and LMIC and (B) Frequency of babies admitted to NICU

| Study | Year | Population | Location of study | Type of study | Seizure diagnosis | Incidence  (1000/LB) | NBW | LBW | VLBW  (1000/LB) |
| --- | --- | --- | --- | --- | --- | --- | --- | --- | --- |
| Holden et al.[5] | 1959–1966 | - | USA, National Collaborative Perinatal Project | Prospective | Clinical | 5.0 | - | 31 | - |
| Lanska et al.[6] | 1985–1989 | Term & preterm  (n=16,428) | Fayette country, Kentucky | Retrospective | Clinical/ EEG (Record review) | 3.5 | 2.8 | 4.4 | 57.5 |
| Lanska and Lanska[7] | 1980–1991 |  | USA, National Hospital Discharge Survey | Retrospective | Clinical | 2.84 | 2.4 | 9.4 | - |
| Saliba et al.[8] | 1992–1994 | Term & preterm  (*n* = 116, 48) | Harris Country, Texas | Retrospective/Prospective | Clinical (Record review) | 1.8 | 1.4 | 5.6 | 19 |
| Ronen et al.[9] | 1990–1995 | Term & preterm | Newfoundland, Canada | Prospective | Clinical/  cEEG | 2.6 | 2.0 | 13.5 | - |
| Glass et al.[10] | 1999–2002 |  | California | Retrospective | Clinical | 0.95 | - | - | NA |
| Pisani et al.[11] | 2002–2014 | Term & preterm  (*n* = 112) | Italy | Retrospective | Electroclinical/ Electrographic | 2.29 | 1.19 |  | 127.57 |
| Mwaniki et al.[12] | 2003–2007 | Term & preterm  (*n* = 1600) | Kenya | Prospective | Clinical | 39.5 (extrapolated) | - | - | - |

| Study | Year | Population | Location of study | Type of study | Seizure diagnosis | Frequency in neonates admitted to NICU | NBW | LBW | VLBW  (1000/LB) |
| --- | --- | --- | --- | --- | --- | --- | --- | --- | --- |
| Shah et al.[13] | 2007–2008 | Term & preterm  (*n* = 51) | UK | Prospective | aEEG | 22% (aEEG0  4% (clinically) | - | - | - |
| Sadeghian et al.[14] | 2007–2009 | Term & preterm  (*n* = 699) | Iran | Case control study | Clinical | 3.6% | - | - | - |
| Ghanshyambhai et al.[15] | 2011–2013 | Term & preterm  (*n* = 10724) | India | Prospective | Clinical | 1.6% | - | - | - |
| Sabzehei et al.[16] | 2008–2011 | Term & preterm  (*n* = 1112) | Iran | retrospective | Clinical | 9.1% | - | 16.7 | 2.9% |
| Mwaniki et al.[12] | 2003–2007 | Term & preterm  (*n* = 1600) | Kenya | Prospective | Clinical | 9% | - | - | - |

**Supplementary Table S2** Etiology of neonatal seizures

|  | **Study** | **Year** | **Duration** | **Numbers** | **Etiology** | | | | | | | | |
| --- | --- | --- | --- | --- | --- | --- | --- | --- | --- | --- | --- | --- | --- |
|  |  |  |  |  | HIE | Stroke | ICH | Metabolic / Electrolyte disturbances | Infections | Congenital CNS malformations | IEM | Genetic / Epilepsy syndromes | Unknown |
| HIC | Glass et al.[17] | 2016 | 2013-2015 | *N* = 426 | 38% | 18% | 12% | 4% | 4% | 4% | 4% | 9% | 9% |
|  | Weeke et al.[18] | 2015 | 2009-2013 | *N* = 378 | 46% | 11% | 12% | 5% | 7% | 3% | 4% | 2% | 9% |
|  | Loman et al.[19] | 2014 | 2002-2009 | *N* = 221 | 59% | 8% | 9% | 11% | 6% | 3% | 2% | 2% | 1% |
|  | Pisani et al.[20] | 2007 | 1999-2000 | *N* = 106 | 43% | - | 24% | 7% | 8% | 6% | 6% | - | 7% |
|  | Tekgul et al.[21] | 2006 | 1997-2000 | *N* = 106 | 40% | 18% | 17% | 3% | 3% | 5% | 1% | - | 12% |
|  | Ronen et al.[9] | 1999 | 1990-1994 | *N* = 89 | 40% | 7% | 11% | 19% | 20% | 10% | - | 6% | 14% |
| LMIC | Nair et al.[22] | 2020 | (1 year) | *N* = 75 | 52% | - | 6% | 16% | 20% | - | - | - | - |
|  | Reddy et al.[23] | 2018 | 2016-2017 | *N* = 84 | 33% | - | 5% | 25% | 25% | 5% | - | - | - |
|  | Ghanshyambhai et al.[24] | 2016 | 2011-2013 | *N* = 172 | 78% | - | 2% | 9% | - | - | - | - | - |
|  | Sadeghian et al.[14] | 2012 | 2007-2009 | *N* = 25 | 32%* |  | 4% | 4% | 8% | - | 4% | - | - |
|  | Holanda et al.[25] | 2006 | 2001-2002 | *N* = 104 | 54% | - | 13% | 32% | 1% | 5% | 1% | - | - |

*HIC* High income countries; *HIE* Hypoxic–ischemic encephalopathy; *IEM* Inborn error of metabolism; *LMIC* Low- and middle-income countries

Percentage may not add to 100% due to rounding, or data provided by original study.

**Supplementary Table S3** Types of clinical seizures and presentation^2^

| **Type** | **Description** | **Special considerations** | **Clinical context of seizure type** |
| --- | --- | --- | --- |
| Automatism | A more or less coordinated motor activity usually occurring when cognition is impaired. This often resembles a voluntary movement and may consist of an inappropriate continuation of preictal motor activity | Typically, oral in neonates. Behavior in term and preterm infants may mimic ictal automatisms, thus EEG/aEEG mandatory. | Seen in HIE and preterm infants. Often part of sequential seizures. |
| Clonic | Jerking, either symmetric or asymmetric, that is regularly repetitive (typically 2-3 Hz) and involves the same muscle groups. | Seizure type, which is more reliably diagnosed clinically. Focal in neonates. | Typical seizure type in neonatal stroke or cerebral hemorrhage. May be seen in HIE and other etiologies. |
| Epileptic spasms | A sudden flexion, extension, or mixed extension–flexion of predominantly proximal and truncal muscles that is usually more sustained than a myoclonic movement but not as sustained as a tonic seizure. | Brief in neonates thus may be difficult to differentiate from myoclonic seizures without an EMG channel. May occur in clusters. | Rare. May be seen in inborn errors of metabolism or early infantile DEE. |
| Myoclonic | A sudden, brief (<100 msec) involuntary single or multiple contraction(s) of muscles(s) or muscle groups of variable topography (axial, proximal limb, distal). | Clinically difficult to differentiate from non-epileptic myoclonus; requires EEG, ideally with EMG channels. | Typical seizure type in inborn errors of metabolism and preterm infants. May also be seen in early-infantile DEE |
| Tonic | A sustained increase in muscle contraction lasting a few seconds to minutes. | Focal, unilateral or bilateral asymmetric. Generalized tonic posturing not of epileptic origin | Typical seizure type early infantile DEE and genetic neonatal epilepsies. |
| Autonomic | A distinct alteration of autonomic nervous system function involving cardiovascular, pupillary, gastrointestinal, sudomotor, vasomotor, and thermoregulatory functions. | May involve respiration (apnea). EEG/aEEG mandatory. | Rare in isolation. Seen in intraventricular hemorrhage as well as temporal or occipital lobe lesions. Also described in early- infantile DEE. |
| Behavioral arrest | Arrest (pause) of activities, freezing, immobilization, as in behavior arrest seizure. | EEG/aEEG mandatory | Rare as an isolated seizure type. More commonly seen as part of the sequential seizure |
| Sequential seizure | This term is used in the instruction manual for the ILAE 2017 operational classification of seizure types for events with a sequence of signs, symptoms, and EEG changes at different times.6 | No predominant feature can be determined, instead, the seizure presents with a variety of clinical signs. Several features typically occur in a sequence, often with changing lateralization within or between seizures. | Often seen in genetic epilepsies such as self-limited neonatal epilepsy or KCNQ2 encephalopathy. |
| Electrographic-only seizure | Subclinical, without clinical manifestation. | EEG/aEEG mandatory | Often seen in preterm infants, HIE (particularly in those with basal ganglia/thalamus injury), critically ill and neonates undergoing cardiac surgery. |
| Unclassified seizure type | Due to inadequate information or unusual clinical features with an inability to place in other categories | EEG/aEEG mandatory |  |

(reprinted with permission from ^2^)

**References**

1. Pellegrin S, Munoz FM, Padula M, et al; Brighton Collaboration Neonatal Seizures Working Group. Neonatal seizures: case definition & guidelines for data collection, analysis, and presentation of immunization safety data. Vaccine. 2019;37:7596–609.

2. Pressler RM, Cilio MR, Mizrahi EM, et al. The ILAE classification of seizures and the epilepsies: modification for seizures in the neonate. Position paper by the ILAE task force on neonatal seizures. Epilepsia. 2021;62:615–28.

3. Mastrangelo M, Cesario S. Update on the treatment of vitamin B6 dependent epilepsies. Expert Rev Neurother. 2019;19:1135–47.

4. Sharma S, Prasad AN. Inborn errors of metabolism and epilepsy: current understanding, diagnosis, and treatment approaches. Int J Mol Sci. 2017;18:1384.

5. Holden KR, Mellits ED, Freeman JM. Neonatal seizures. I. Correlation of prenatal and perinatal events with outcomes. Pediatrics. 1982;70:165–76.

6. Lanska MJ, Lanska DJ, Baumann RJ, Kryscio RJ. A population-based study of neonatal seizures in Fayette County, Kentucky. Neurology. 1995;45:724–32.

7. Lanska MJ, Lanska DJ. Neonatal seizures in the United States: results of the national hospital discharge survey, 1980–1991. Neuroepidemiology. 1996;15:117–25.

8. Saliba RM, Annegers JF, Waller DK, Tyson JE, Mizrahi EM. Incidence of neonatal seizures in Harris County, Texas, 1992–1994. Am J Epidemiol. 1999;150:763–9.

9. Ronen GM, Penney S, Andrews W. The epidemiology of clinical neonatal seizures in Newfoundland: a population-based study. J Pediatr. 1999;134:71–5.

10. Glass HC, Pham TN, Danielsen B, Towner D, Glidden D, Wu YW. Antenatal and intrapartum risk factors for seizures in term newborns: a population-based study, California 1998-2002.  J Pediatr. 2009;154:24–8.

11. Pisani F, Facini C, Bianchi E, Giussani G, Piccolo B, Beghi E. Incidence of neonatal seizures, perinatal risk factors for epilepsy and mortality after neonatal seizures in the province of Parma, Italy. Epilepsia. 2018;59:1764–73.

12. Mwaniki M, Mathenge A, Gwer S, et al. Neonatal seizures in a rural Kenyan District Hospital: aetiology, incidence and outcome of hospitalization. BMC Medicine. 2010;8:16.

13. Shah DK, Zempel J, Barton T, Lukas K, Inder TE. Electrographic seizures in preterm infants during the first week of life are associated with cerebral injury. Pediatr Res. 2010;67:102–6.

14. Sadeghian A, Damghanian M, Shariati M. Neonatal seizures in a rural Iranian district hospital: etiologies, incidence and predicting factors. Acta Med Iran. 2012;50:760–4.

15. Ghanshyambhai P, Sharma D, Patel A, Shastri S. To study the incidence, etiology and EEG profile of neonatal seizures: a prospective observational study from India. TJ Matern Fetal Neonatal Med. 2016;29:554–8.

16. Sabzehei MK, Basiri B, Bazmamoun H. The etiology, clinical type, and short outcome of seizures in newbornsHospitalized in Besat Hospital/Hamadan/ Iran.  Iran J Child Neurol. 2014;8:24–8.

17. Glass HC, Shellhaas RA, Wusthoff CJ, et al. Contemporary profile of seizures in neonates: a prospective cohort study. J Pediatr. 2016;174:98–103.

18. Weeke LC, Groenendaal F, Toet MC, et al. The aetiology of neonatal seizures and the diagnostic contribution of neonatal cerebral magnetic resonance imaging. Dev Med Child Neurol. 2015;57: 248–56.

19. Loman AM, ter Horst HJ, Lambrechtsen FA, Lunsing RJ. Neonatal seizures: aetiology by means of a standardized work-up.  Eur J Paediatr Neurol. 2014;18:360–7.

20. Pisani F, Cerminara C, Fusco C, Sisti L. Neonatal status epilepticus vs recurrent neonatal seizures: clinical findings and outcome. Neurology. 2007;69:2177–85.

21. Tekgul H, Gauvreau K, Soul J, et al. The current etiologic profile and neurodevelopmental outcome of seizures in term newborn infants. Pediatrics. 2006;117:1270–80.

22. Nair B, Sharma J, Chaudhary S. Clinicoetiological profile of neonatal seizure in a newborn care unit of a tertiary care teaching hospital in Northern India. J Clin Neonatol. 2020;9:27–31.

23. Reddy K, Soren C, Jagtap S, Pardhasaradhi Y, Satish S. Clinico-etiological profile of neonatal seizures in term neonates. . Int J Contemp Pediatr. 2018;7:211.

24. Ghanshyambhai P, Sharma D, Patel A, Shastri S. To study the incidence, etiology and EEG profile of neonatal seizures: A prospective observational study from India. J Matern Fetal Neonatal Med. 2016;29:554–8.

25. Holanda MR, Melo AN. Comparative clinical study of preterm and full-term newborn neonatal seizures. Arq Neuropsiquiatr. 2006;64:45–50.
